# Aqueous solubilization of C_60_ fullerene by natural protein surfactants, latherin and ranaspumin-2

**DOI:** 10.1016/j.bpc.2016.05.003

**Published:** 2016

**Authors:** Steven J. Vance, Vibhuti Desai, Brian O. Smith, Malcolm W. Kennedy, Alan Cooper

**Affiliations:** a School of Chemistry, University of Glasgow, Glasgow, G12 8QQ, UK; b Institute of Molecular, Cell & Systems Biology, College of Medical, Veterinary and Life Sciences, University of Glasgow, G12 8QQ, UK; c Institute of Biodiversity, Animal Health and Comparative Medicine, College of Medical, Veterinary and Life Sciences, University of Glasgow, G12 8QQ, UK

**Keywords:** Rsn-2, ranaspumin-2, PLUNC, palate, lung, and nasal epithelium clone protein, SWNT, single-wall nanotubes, BSA, bovine serum albumin

## Abstract

C_60_ fullerene is not soluble in water and dispersion usually requires organic solvents, sonication or vigorous mechanical mixing. However, we show here that mixing of pristine C_60_ in water with natural surfactant proteins latherin and ranaspumin-2 (Rsn-2) at low concentrations yields stable aqueous dispersions with spectroscopic properties similar to those previously obtained by more vigorous methods. Particle sizes are significantly smaller than those achieved by mechanical dispersion alone, and concentrations are compatible with clusters approximating 1:1 protein:C_60_ stoichiometry. These proteins can also be adsorbed onto more intractable carbon nanotubes. This promises to be a convenient way to interface a range of hydrophobic nanoparticles and related materials with biological macromolecules, with potential to exploit the versatility of recombinant protein engineering in the development of nano-bio interface devices. It also has potential consequences for toxicological aspects of these and similar nanoparticles.

## Highlights

• C_60_ solubilized in water by mixing with natural surfactant proteins.
• Aqueous fullerene: protein solutions have long term stability.
• C_60_ cluster sizes are smaller than those obtained by more vigorous methods.
• Potential implications for nanoparticle toxicology and biotechnology

## Graphical abstract

**Figure:**
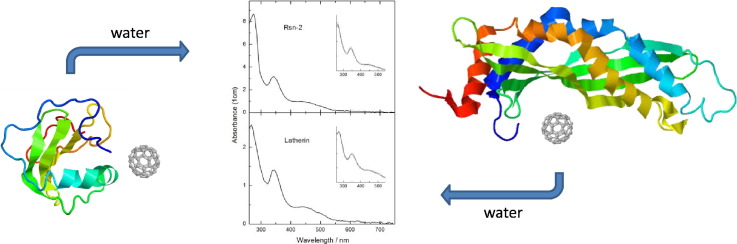

## 1 Introduction

Despite much initial promise, exploitation of fullerenes and other carbon-based nanomaterials in aqueous systems has been frustrated by the inevitable insolubility of such materials in water. In the course of our ongoing studies of natural surfactant proteins from frogs and horses [1], [2], [3], [4], [5], [6] it occurred to us that solubilization or dispersion of fullerenes in water might be facilitated by interaction with such materials. Here we report on preliminary observations that indicate that this is indeed the case.

**Fig. 1:**
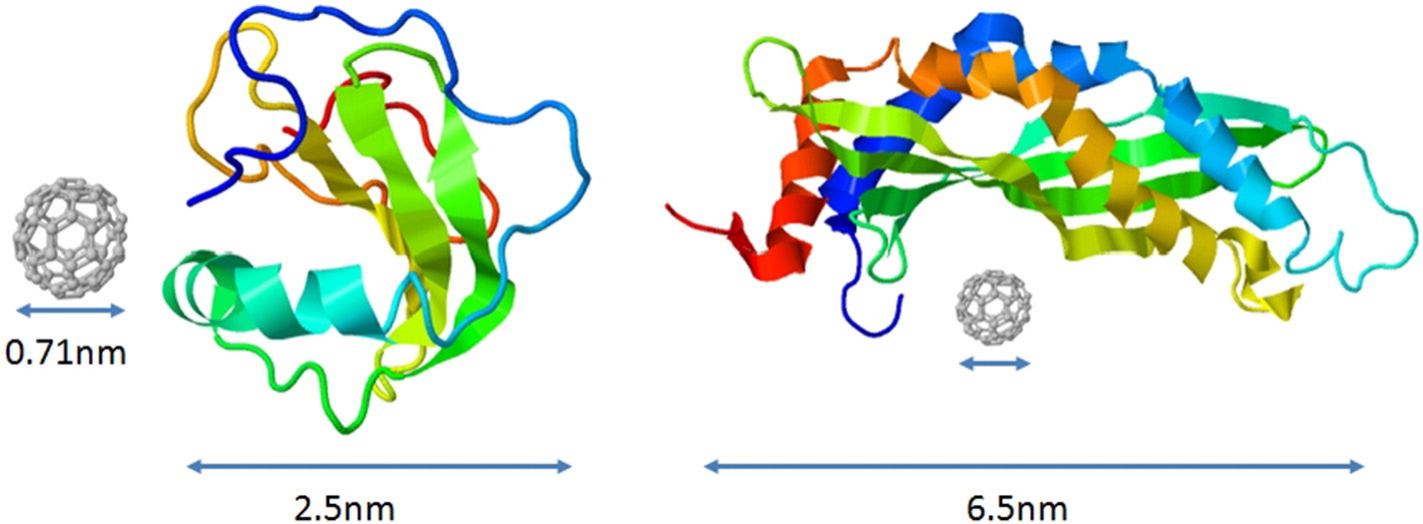
Cartoon illustrating the size of C_60_ in comparison to the surfactant proteins Rsn-2 (mw 13,415 Da; left) and latherin (mw 22,899 Da; right), approximately to scale. The structures for Rsn-2 (pdb: 2WGO) and latherin (pdb: 3ZPM) are taken from [4] and [6], respectively.

Ranaspumin-2 (Rsn-2) and latherin are potent surfactant proteins found in the foam nests of a species of tropical frog and horse sweat and saliva, respectively [1], [7]. In its natural biological context, Rsn-2 is one of the proteins responsible for reducing aqueous surface tension to facilitate biocompatible foam nest formation, whereas latherin appears to act as a wetting agent to aid evaporative cooling in horses. These otherwise unrelated proteins are monomeric in solution, with structures that are typical of small water-soluble globular proteins (see Fig. 1), as determined by high-resolution protein NMR techniques [4], [6]. Unusually however, for proteins in their native state, both have a strong propensity to adsorb onto hydrophobic surfaces or to partition into the air-water interface layer in ways consistent with their biological function [1]. Studies using small-angle neutron reflection measurements suggest that this involves relatively modest conformational changes which, at least in the case of Rsn-2, retain protein secondary structure, as shown by surface IR methods [4]. These proteins are highly soluble in their native state, with dimensions commensurate with possible interaction with fullerene molecules (Fig. 1). The van der Waals diameter of a C_60_ molecule is about 1.01 nm, with a nucleus to nucleus diameter of about 0.71 nm — small enough to fit inside the hydrophobic protein compartments that might be formed by modest hinge-bending conformational change, as envisaged for example in the case of Rsn-2 [4].

For these initial exploratory studies we have examined the effects upon mixing of C_60_ fullerene in aqueous solutions of the surfactant proteins latherin and Rsn-2, together with control experiments using other proteins and cyclodextrins for comparison. Samples were analysed by UV–vis and NMR spectroscopy, together with protein concentration analysis where appropriate. We find that these two surfactant proteins do indeed interact with and disperse C_60_ fullerene into the aqueous phase, and that they may also interact with carbon nanotubes.

## 2 Experimental

Materials: recombinant proteins Rsn-2 and latherin, both natural abundance and ^15^N-enriched for NMR, were prepared, purified and characterised as previously described [3], [4], [5], [6]. Control proteins and other reagents were obtained from commercial sources as follows, and used as received: fullerene-C_60_ (Sigma-Aldrich 483036), single-wall nanotubes (SWNT) (Sigma-Aldrich 704121; carbon nanotube, single-walled, 0.7–1.1 nm diameter), hen egg white lysozyme (Sigma L-6876), bovine serum albumin (BSA; Sigma A-6003), β-cyclodextrin (Sigma C-4767), hydroxypropyl-β-cyclodextrin (Aldrich 33,260-7). Natural frog foam fluid containing a natural cocktail of proteins and carbohydrate components was isolated by centrifugation from nest foams of the tropical túngara frog *Engystomops*(=* Physalaemus*) *pustulosus*, as described in [2], [3]. Aqueous samples were dissolved either in deionized water (cyclodextrins) or in 20 mM sodium phosphate/50 mM NaCl buffer, pH 7.5 (proteins).

Sample preparation: typically, about 10 mg of solid C_60_ was suspended in 1–2 ml of buffer containing the test protein (1–7 mg/ml), or control, in a 7 ml polystyrene bijou tube, and stirred for several days (1–15 days) at 4 °C using a magnetic stirrer with ca. 5 mm stir bars. The stirring speed was adjusted to be fast enough to keep solid particles in suspension, but to avoid foaming. Aliquots were taken periodically at intervals for UV–vis spectroscopic analysis using a NanoDrop ND-1000 spectrophotometer (Thermo Scientific, USA) 0.1 or 1 mm path length, following brief centrifugation (Heraeus Biofuge Fresco bench microfuge, 16,000 ×* g*) to remove undissolved material.

Protein concentrations in solution were determined from the 280 nm UV absorbance using molar extinction coefficients, ε_280_ (1 cm), 6085 (Rsn-2, mw 13,415) and 10,095 (latherin, mw 22,899). For samples in which the 280 nm protein absorbance was masked by other components (C_60_, etc.), the protein concentration was measured using a standard colorimetric method (Bio-Rad Inc. “Quick Start™” Bradford Protein Assay), calibrated using standard Rsn-2 or latherin solutions, as appropriate.

NMR: Protein-C_60_ solutions were monitored by solution NMR spectroscopy to examine the state of the protein. The ^15^N-labelled Rsn-2 mixed with C_60_ as described above was analysed by 1D ^1^H double pulsed field gradient spin echo [8] and 2D ^15^N,^1^H heteronuclear single quantum correlation (HSQC) spectroscopy [9] at 14.1 Tesla using a Bruker AVANCE spectrometer equipped with a TCI cryoprobe, with ^15^N-Rsn-2 concentrations of up to 530 μM.

## 3 Results & discussion

**Fig. 2:**
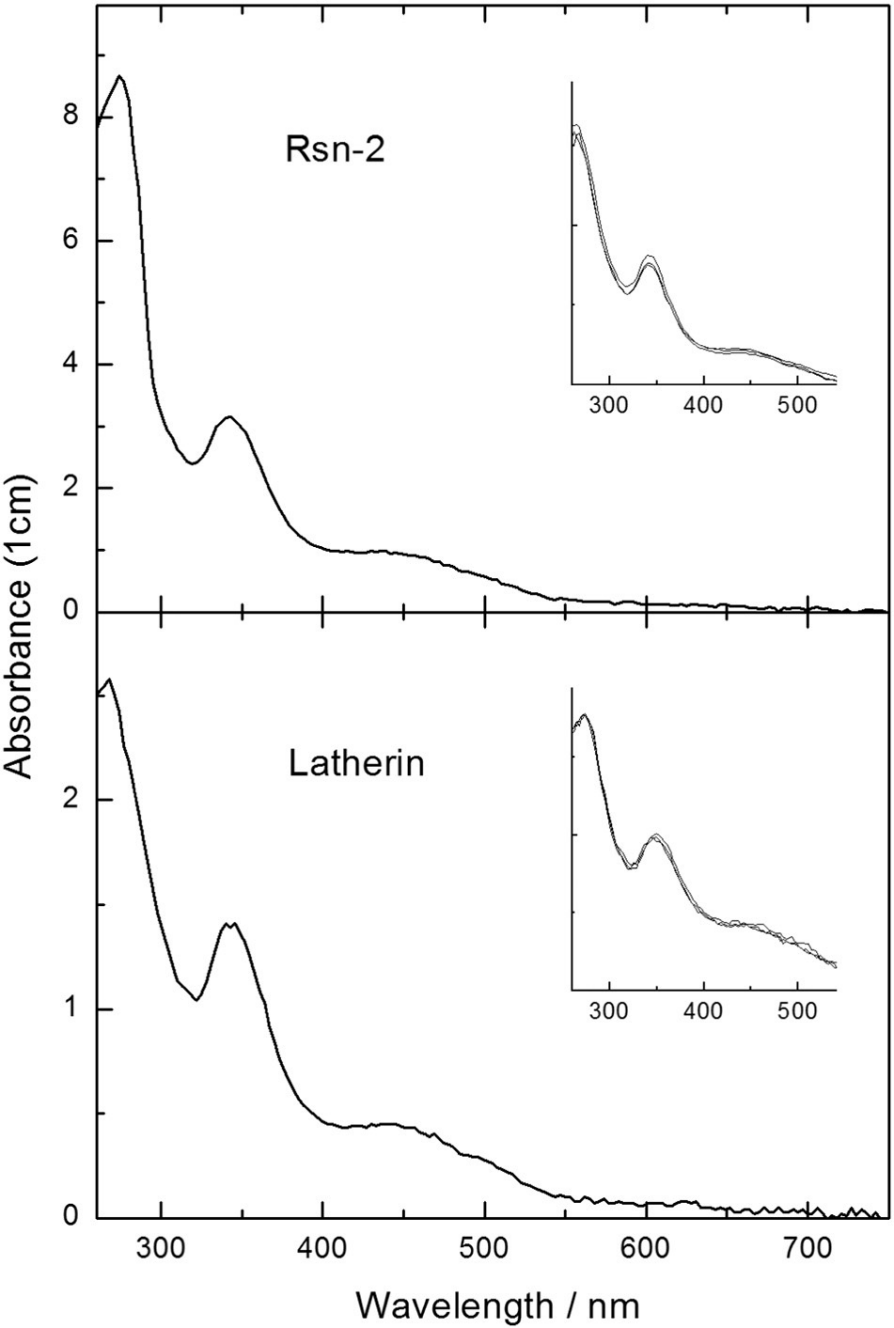
Examples of UV–vis spectra of C_60_ dispersed in aqueous solutions of Rsn-2 (upper panel) or latherin (lower panel). The inserts show superimposed spectra of samples after storage at 4 °C for approximately 1, 2 and 6 months. (Absorbances measured on a Nanodrop using 0.1 or 1 mm path length, but scaled here to 1 cm path length for convenience.)

Stirring of solid pristine C_60_ in dilute aqueous solutions of either latherin or Rsn-2 (1–7 mg/ml) gave pale brown suspensions typical of the fullerene dispersions produced by more aggressive techniques such as grinding or sonication [10], [11]. After centrifugation to remove excess C_60_ and other particulate matter, the supernatant was water-clear and straw-coloured with prominent UV–vis absorbance bands around 342 nm together with a much broader peak in the 440–460 nm range (Fig. 2; Table 1), as is typically observed for aqueous dispersions of C_60_ produced by other methods [11], [12], [13], [14], [15], [16], [17], [18]. For comparison, control experiments using β-cyclodextrin or hydroxypropyl-β-cyclodextrin solutions (2% w/v) under the same conditions gave clear supernatants with C_60_ absorbance bands at 263(± 3), 343(± 3) and 445(± 5) nm, as previously reported [11], [15], [16]. Note that the shorter wavelength band is obscured in the latherin/Rsn-2 experiments by the intrinsic UV absorbance of the proteins in this region. No aqueous dispersion of C_60_ was seen in the absence of additives under these relatively gentle stirring conditions.

As commonly observed with heterogeneous particulate materials such as these, the rates and extents of dispersion can depend unpredictably on numerous physical factors such as stirrer shape, speed of stirring, etc. However, in our experiments, solution/dispersion with latherin or Rsn-2 was usually apparent within 1–5 days under most circumstances.

Control experiments involving mixing of C_60_ with marginally surfactant or non-surfactant proteins (BSA and lysozyme, respectively) gave erratic and unpredictable results. As described previously by others [19], [20], BSA did give some dispersion but, in our hands under these conditions, this was generally transient and the mixtures rapidly coagulated and precipitated, leaving supernatants devoid of both C_60_ and BSA, as determined by UV absorbance and colorimetric protein analysis (Bradford assay). This is consistent with the surface denaturation or partial unfolding of BSA thought to be the basis for the non-specific surface coating properties of such marginally surfactant proteins [20], [21], [22]. Similar variability involving transient dispersion and precipitation was observed with lysozyme, with no stable C_60_ dispersions obtained.

These observations with BSA and lysozyme were in marked contrast to our results with natural surfactant proteins, where the C_60_ solutions in Rsn-2 or latherin mixtures remained water-clear, stable and spectroscopically unchanged for long periods of time – up to six months in trials so far – when stored at 4 °C (see Fig. 1). Gel electrophoresis (SDS-PAGE, data not shown) showed no protein degradation in the presence of C_60_ under similar conditions. Analysis of the protein content of the C_60_ supernatants (using the Bradford assay) showed that most of the Rsn-2 or latherin (> 90%) remained in solution and was not co-precipitated in the excess C_60_ pellet. Since denatured/unfolded proteins are generally very sticky and insoluble, this suggests that the natural surfactant proteins could retain significant native structure under these conditions, as we have observed elsewhere as part of their natural surfactant activities [1], [3], [5]. We have shown, for example, that latherin in dilute aqueous solution adheres strongly to a non-polar surface, whereas lysozyme does not under the same mild conditions [5]. It appears to be characteristic of natural surfactant proteins that interaction with non-polar interfaces can be accomplished by relatively subtle conformational change, rather than by more disruptive denaturation.

Mechanical dispersion of fullerene in water usually results in heterogeneous mixtures of C_60_ aggregates with a wide range of particle sizes, and with spectral properties that depend upon the particle size distribution [12], [13], and this allows us to estimate possible limits on the C_60_ cluster sizes that we observe here. Careful studies elsewhere have shown that there is an empirical relationship between C_60_ cluster size and near-UV absorbance wavelength [10], [12], [13]. In particular, the position of the near-UV peak ranges from 350 to 360 nm in aqueous C_60_ clusters produced by mechanical dispersion, and is strongly correlated with cluster size, with an extrapolated λ_max_ of around 340 nm for the hydrated monomer. For the range of Rsn-2-C_60_ samples that we have examined so far, we observed absorbance λ_max_ values of 342 nm (± 2 nm; n = 16), approaching what might be expected for a hydrated C_60_ monomer [10], [13]. We saw slightly longer λ_max_ values for latherin-C_60_ samples at 346 nm (± 3 nm; n = 4), suggesting slightly larger C_60_ clusters (or less solvated environment) with this protein. For comparison, single experiments with cyclodextrins yield λ_max_ values of 346 and 340 nm for β-cyclodextrin and hydroxypropyl-β-cyclodextrin solutions, respectively (Table 1).

Interestingly, for transient BSA-C_60_ solutions prior to spontaneous precipitation, absorbance λ_max_ was about 352 nm, consistent with the larger clusters that might be anticipated here for dispersion involving non-specific protein denaturation and aggregation. This, however, apparently conflicts with previous studies reporting stable BSA-C_60_ dispersions with λ_max_ of about 343 nm [20]. We are currently unable to account for this discrepancy, but it seems to reflect inherent difficulties commonly experienced with replication in these systems.

**Fig. 3:**
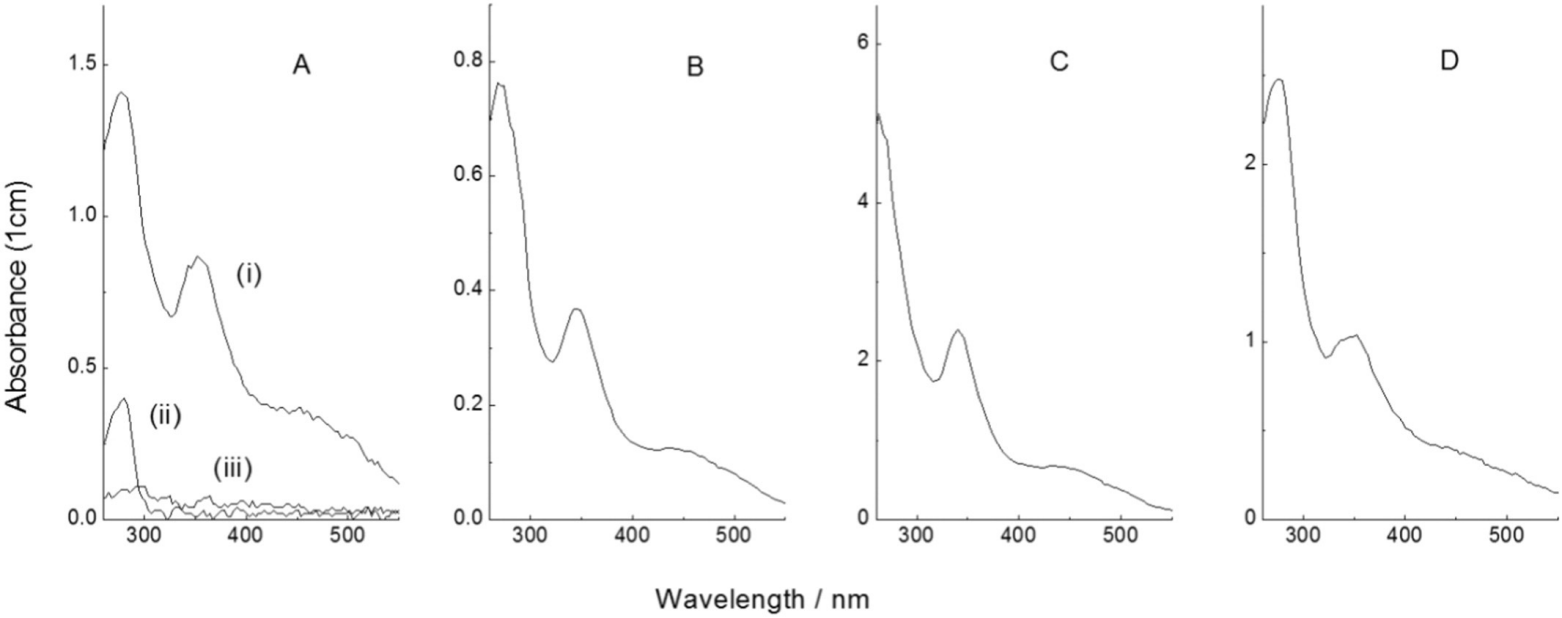
Examples of UV–vis spectra of C_60_ dispersed in aqueous solutions of: (A) BSA; (B) lysozyme; (C) hydroxypropyl-β-cyclodextrin; (D) natural frog foam nest fluid. Note that the BSA and lysozyme samples are unstable and precipitate irreversibly shortly after formation. This is illustrated in panel (A), where line (i) is the spectrum of the BSA-C_60_ dispersion after 1 day mixing, together with the spectrum of the BSA solution alone (ii) showing the typical 280 nm protein absorbance band. Both these bands disappear after spontaneous precipitation (iii) a few days later.

Additional particle size information can be gleaned from other aspects of the UV–vis absorbance spectra (Fig. 2, Fig. 3). In particular, it is clear that the subsidiary absorbance band around 450 nm is significantly less pronounced here than is observed for larger C_60_ clusters produced by mechanical dispersion in the absence of additives [13]. Empirical data show a strong correlation between C_60_ cluster size and the A_450_/A_360_ absorbance band amplitude ratios, with A_450_/A_360_ values increasing from 0.4 to 0.9 over the 120–400 nm hydrodynamic diameter range accessible from simple mechanical dispersion [12], [13]. As shown in Table 1, A_450_/A_360_ absorbance ratios for the natural surfactant protein dispersions presented here are significantly lower (consistent with cluster diameters probably much smaller than 100 nm) and smaller than the clusters formed by most other techniques [11], [15]. However, some caution is warranted here because the correlations in absorbance properties reported for mechanical dispersions [10], [12], [13] relate (presumably) to fully hydrated C_60_ clusters, whereas our observations are for clusters stabilised by interaction with protein. This may affect spectral properties of the clusters that are hard to predict at this stage.

We have no direct evidence yet for the state of the C_60_ molecules in the latherin/Rsn-2 preparations, but one can propose various ways in which surfactant proteins might assist solubilization or dispersion in water. This might include the formation of specific stoichiometric (1:1) protein-C_60_ monomeric complexes or, alternatively, more heterogeneous C_60_ clusters stabilised by multiple protein interactions, either acting as a glue or coat for larger clusters in the aqueous environment. However, the existence of very large protein-C_60_ aggregates seems to be ruled out by persistence of the protein-C_60_ in solution, and the optical clarity of the solutions, as well as by the UV–vis spectral properties described above.

The stoichiometries of the soluble protein-C_60_ complexes are difficult to estimate precisely from spectral data because of major uncertainties in the UV–vis molar extinction coefficients of C_60_ in aqueous dispersions. Literature values for the characteristic 360 nm band vary widely over the range 30,000–60,000 M^− 1^ cm^− 1^ for ε_360_ in water [11], [12], [13], [14], [18], with much of this variability arising from the different C_60_ cluster sizes obtained by different methods of preparation [12]. Consequently, it is possible only to make order-of-magnitude estimates of relative concentrations here. For purposes of estimation, we have assumed a representative ε_360_ value of 60,000 M^− 1^ cm^− 1^ appropriate for small C_60_ clusters [12] in order to estimate solubilized C_60_ concentrations. In a series of experiments involving Rsn-2 concentrations in the range 1–7 mg/ml (75–530 μM), with incubation/mixing times of 3–15 days, the molar C_60_ concentrations in solution were always less than or equal to that of the Rsn-2. This is consistent with a stoichiometric C_60_: Rsn-2 ratio not exceeding 1:1 in the clusters.

**Fig. 4:**
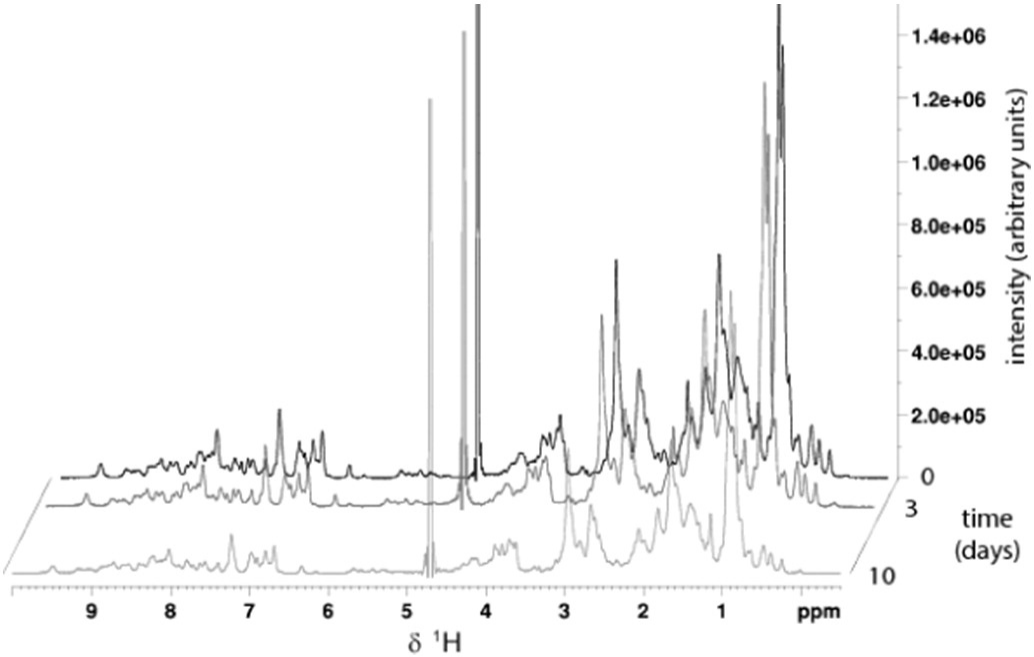
1D ^1^H NMR spectra of Rsn-2 before addition of C_60_ and at 3 and 10 days after incubation with fullerene. Spectra were recorded on ^15^N labelled Rsn-2 using a double pulsed field gradient spin echo pulse sequence incorporating ^15^N decoupling during acquisition and processed with a 90° shifted sinebell squared window function. The signal intensities are reduced by approximately 15 and 45% after 3 and 10 days respectively. (Note: the peak at about 4.7 ppm comes from residual water.)

However, this does not necessarily imply that clusters are monomeric, and does not rule out the possibility of larger protein/C_60_ clusters containing both components in similar proportions. In order to address this question further we have performed solution NMR experiments using ^15^N-enriched Rsn-2 to follow the state of the protein as C_60_ complexes developed. In 1D-NMR and ^15^N-HSQC experiments with Rsn-2/C_60_ samples (Fig. 4) we observed that the positions and linewidths of protein NMR peaks were unaltered, but their intensities were uniformly decreased (by ~ 15% after ~ 3 days and ~ 45% after ~ 10 days) showing that a proportion of the protein remained structured and monomeric in solution, while the remainder became invisible to solution NMR in the presence of C_60_ under these conditions. This also confirms the lack of chemical degradation of Rsn-2. UV–vis analysis of the same samples indicated that, with a ^15^N-Rsn-2 starting concentration of 530 μM, the C_60_ concentration in solution increased to 45 μM (3 days) and 280 μM (10 days), respectively. Assuming 1:1 C_60_: Rsn-2 stoichiometry, this corresponds to roughly 10% and 50% of the protein involved in C_60_ clusters at 3 and 10 days, respectively. These values are of similar magnitude to those relating to loss of NMR peak intensities in the same samples, indicating that the C_60_/Rsn-2 clusters are probably larger than monomeric, with aggregate molecular weights of at least 100 kDa, resulting in long tumbling times and linewidth broadening that make them invisible to NMR.

A cluster size of (say) 10 C_60_ molecules, each together with an accompanying Rsn-2 molecule (mw 13.4 kDa) would tumble too slowly in solution to be visible by NMR, yet would still be soluble and small enough to give the observed UV–vis spectral properties consistent with extrapolated values for small clusters [10], [13].

**Fig. 5:**
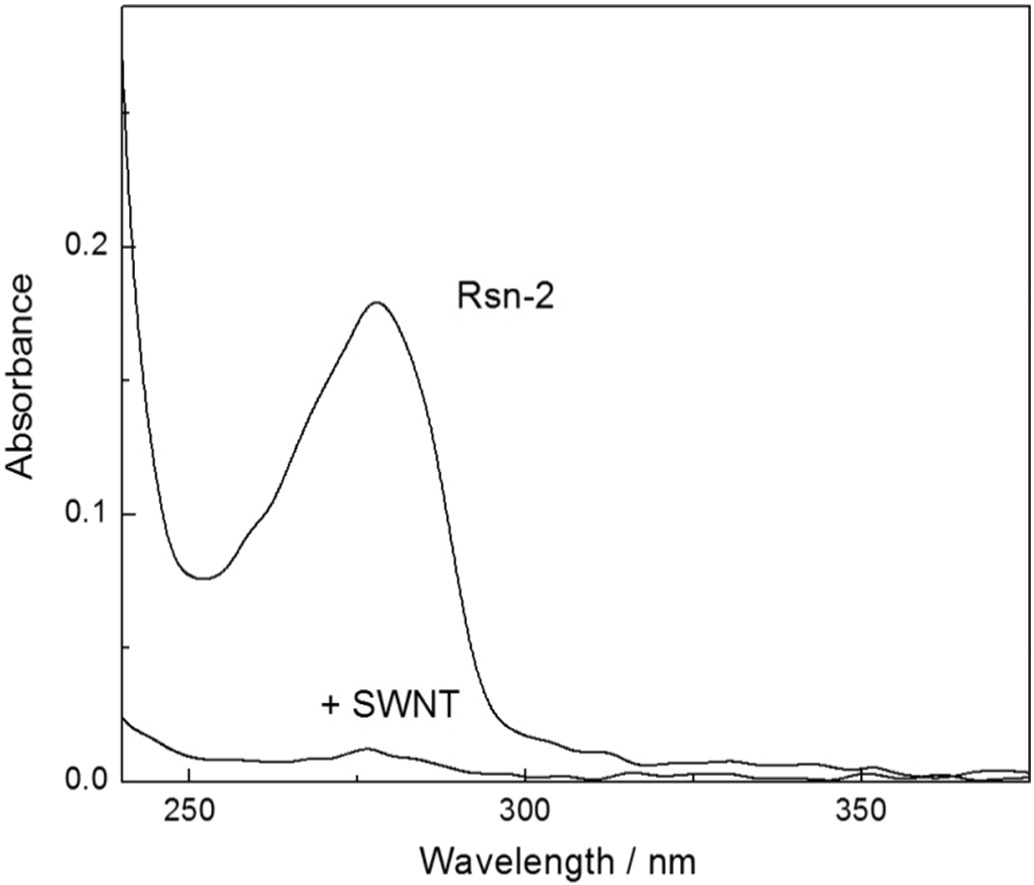
UV–vis spectra illustrating the loss of the characteristic protein absorbance of Rsn-2 upon incubation with carbon nanotubes (SWNT).

Having demonstrated significant protein-mediated solubilization of fullerene with Rsn-2 and latherin, it is tempting to examine what might happen with other carbon allotropes of interest. Although we have not (yet) observed true solubilization, our preliminary trials with single-wall carbon nanotubes (SWNT) in water look promising. Full solubilization of SWNT with surfactant proteins might be unlikely given the large size, rod-like aspect ratio and size heterogeneity of the nanotubes, but we found that mixing SWNT with either of our proteins brings about significant changes in the suspension's visual appearance (black to pale-grey turbid suspension) that are characteristic of nanotube dispersion in water [23]. This is accompanied by total loss of protein from the supernatant (Fig. 5) upon centrifugation, consistent with adsorption of the protein onto nanotubes.

In view of potential environmental concerns, and in light of projected large-scale industrial production and application of fullerenes and related materials together with the inevitable contamination issues, it is important to explore the ways in which such materials may interact and disperse in the wider natural environment. Although the particular proteins we deal with here are unlikely to represent significant vehicles for the environmental dispersal of C_60_-like nanoparticles, their behaviour may exemplify such effects with natural surfactant proteins from more widespread sources. Such proteins may occur more widely in nature than currently realised [1], [24], and will likely occur in complex mixtures. One of the proteins that we have been using here (Rsn-2) is merely one component in a cocktail of ranaspumin proteins that we have identified in the foam nest fluids of a tropical frog [3]. This fluid, collected in the field as part of other projects [2], [3], [4], gives very similar C_60_ dispersion to that obtained using recombinant Rsn-2 on its own. Although Rsn-2 is just one component in this cocktail, mixing of the natural foam fluid with C_60_ leads to dispersion/solubilization with UV–vis spectra similar to those obtained with pure recombinant Rsn-2 (Fig. 3, Table 1). Interestingly, judging from the λ_max_ and absorbance ratios, the apparent cluster size might be somewhat larger in this case, possibly because of the much lower protein concentration and the presence of other components in the natural fluid. Bearing in mind the way in which these frogs construct the nests in water, using a rapid “egg-beater” mixing process, it is not hard to envisage how carbon and other nanoparticles might enter the biological milieu, with subsequent uptake into biological cells and systems. These observations also have potential consequences for toxicological aspects of nanoparticles, with particular relevance to considerations of toxicity of fullerenes and carbon nanotubes, where, for example, lung surfactants and the latherin-related PLUNC proteins of the human oral and upper respiratory tract [25], [26], [27], might be significant in the uptake, tissue penetration, and/or clearance of airborne particles.

As indicated by references cited herein, non-specific aqueous dispersion of fullerenes by proteins and protein interactions with nanotubes have been previously reported, and more recent reports show that this can also be achieved by appropriately-designed peptides [28]. In the work we have presented here, we have shown how it can be possible to take advantage of the specific structural and functional properties of natural surfactant proteins (such as latherin and Rsn-2) to effect solubilization and interaction with fullerenes and related materials, avoiding more aggressive sonication or chemical modification techniques that have been employed elsewhere (see [29], [30] for recent examples). This offers a convenient way in which to design biological molecules specifically for interactions with hydrophobic nanoparticles and surfaces. Recombinant protein engineering may further release the potential versatility and flexibility of natural surfactant proteins in the development of nanomolecular/biomolecular interfaces using, for example, fusions of proteins such as latherin and Rsn-2 with enzymes or other proteins with useful properties.

## Author contributions

Original concept: AC.

Sample preparation and experiments: AC, SV, VD.

Data analysis and interpretation: AC, BOS, MWK.

Wrote paper: AC, with input from all co-authors.

## Figures and Tables

**Table 1:** Spectroscopic properties of aqueous C_60_ dispersions.^a^

	UV–vis peaks λ_max/_nm	Absorbance ratio A_450_/A_360_	Notes *Except where indicated, all samples were in aqueous buffer, pH* *7.5*
Rsn-2 (n = 16)	342 (± 2)	0.30 (± 0.04)	Stable for at least 6 months at 4 °C
440–450		Protein concentration range 1–7 mg/ml
Latherin (n = 4)	346 (± 3)	0.38 (± 0.06)	Stable for weeks, with no precipitation
440–460		1–2 mg/ml
β-cyclodextrin (n = 1)	346	0.32	In water (2% w/v)
450		
Hydroxypropyl-β-CD (n = 2)	340	0.27	In water (2% w/v)
440–450		
BSA (n = 1) (*before precipitation*)	352	0.43	Transient — precipitates erratically
450–460		1–10 mg/ml
Lysozyme (n = 1) (*before precipitation*)	344	0.32	Transient — precipitates erratically
450		1 mg/ml
Frog foam fluid (n = 1)	340–350	0.42	Natural mixture of Rsn-2 with a range of other proteins and carbohydrates. Total protein ca. 1 mg/ml, of which roughly 10% is Rsn-2

a n is the number of separate observations. Solid C_60_ present in excess.
